# Supplementary material for: Skyrmionic Polarization Textures in Structured Dielectric Planar Media
Source: Nanophotonics. 2026 Feb 8;15(3):e70009. doi: 10.1002/nap2.70009 (PMC12965000; doi:10.1002/nap2.70009)
Supplement: Supplementary file 1 — Supporting Information S1 [file NAP2-15-e70009-s001.pdf]

SUPPLEMENTARY MATERIAL:
Skyrmionic polarization textures in structured dielectric planar media

Francesco Di Colandrea,[1, *] Lorenzo Marrucci,[1, 2] and Filippo Cardano[1, †]

[1]*Dipartimento di Fisica "Ettore Pancini", Università degli Studi di Napoli Federico II, Complesso Universitario di Monte Sant'Angelo, Via Cintia, 80126 Napoli, Italy*
[2]*CNR-ISASI, Institute of Applied Science and Intelligent Systems, Via Campi Flegrei 34, 80078 Pozzuoli (NA), Italy*

S1. ANALYTICAL EXPRESSIONS OF EIGENVALUES AND EIGENSTATES

We recall the decomposition of Eq. (8), valid for an arbitrary SU(2) unitary operator:

$$U(x,y) = e^{-iE(x,y)\mathbf{n}(x,y)\cdot\boldsymbol{\sigma}} = \cos E(x,y)\,\sigma_0 - i\sin E(x,y)\,(\mathbf{n}(x,y)\cdot\boldsymbol{\sigma})\,, \tag{S1}$$

where σ_0 is the 2×2 identity matrix and $\boldsymbol{\sigma} = (\sigma_1, \sigma_2, \sigma_3)$ is the vector of the Pauli matrices. By using the fact that $\mathrm{Tr}\,(\sigma_\ell \sigma_m) = 2\delta_{\ell m}$, with $\delta_{\ell m}$ the Kronecker delta symbol, we derive the energy eigenvalues E and the three components of the Stokes eigenvectors via the following relations:

$$\begin{aligned} E &= \arccos\left(\frac{1}{2}\mathrm{Tr}\,U\right)\,,\\ n_\ell &= \frac{i}{2\sin E}\mathrm{Tr}\,(U\sigma_\ell)\,, \end{aligned} \tag{S2}$$

with $\ell = \{1,2,3\}$. In Eq. (S2), the dependence of $(E, \mathbf{n})$ on (x, y) has been omitted for simplicity. By substituting in Eq. (S1) the expression of the optical operator U defined in Eq. (2), we obtain

$$\begin{aligned}
E(x',y') &= \arccos\left[\frac{-\sin\left(\frac{\delta_x}{2}\right)\left(\cos(x')\cos\left(\frac{\delta_y}{2}\right)+\cos(x'-y')\sin\left(\frac{\delta_y}{2}\right)\right)+\cos\left(\frac{\delta_x}{2}\right)\left(\cos\left(\frac{\delta_y}{2}\right)-\cos(y')\sin\left(\frac{\delta_y}{2}\right)\right)}{\sqrt{2}}\right],\\
n_1(x',y') &= \frac{-\cos\left(\frac{\delta_x}{2}\right)\left(\cos\left(\frac{\delta_y}{2}\right)+\sin\left(\frac{\delta_y}{2}\right)\cos(y')\right)+\sin\left(\frac{\delta_x}{2}\right)\left(-\cos(x')\cos\left(\frac{\delta_y}{2}\right)+\cos(x'-y')\sin\left(\frac{\delta_y}{2}\right)\right)}{\sqrt{2-\left[\sin\left(\frac{\delta_x}{2}\right)\left(\cos(x')\cos\left(\frac{\delta_y}{2}\right)+\cos(x'-y')\sin\left(\frac{\delta_y}{2}\right)\right)+\cos\left(\frac{\delta_x}{2}\right)\left(-\cos\left(\frac{\delta_y}{2}\right)+\cos(y')\sin\left(\frac{\delta_y}{2}\right)\right)\right]^2}},\\
n_2(x',y') &= \frac{-\cos\left(\frac{\delta_y}{2}\right)\sin\left(\frac{\delta_x}{2}\right)\sin(x')-\sin\left(\frac{\delta_y}{2}\right)\left(\cos\left(\frac{\delta_x}{2}\right)\sin(y')+\sin(x'-y')\sin\left(\frac{\delta_x}{2}\right)\right)}{\sqrt{2-\left[\sin\left(\frac{\delta_x}{2}\right)\left(\cos(x')\cos\left(\frac{\delta_y}{2}\right)+\cos(x'-y')\sin\left(\frac{\delta_y}{2}\right)\right)+\cos\left(\frac{\delta_x}{2}\right)\left(-\cos\left(\frac{\delta_y}{2}\right)+\cos(y')\sin\left(\frac{\delta_y}{2}\right)\right)\right]^2}},\\
n_3(x',y') &= \frac{-\cos\left(\frac{\delta_y}{2}\right)\sin\left(\frac{\delta_x}{2}\right)\sin(x')-\sin\left(\frac{\delta_y}{2}\right)\left(\cos\left(\frac{\delta_x}{2}\right)\sin(y')-\sin(x'-y')\sin\left(\frac{\delta_x}{2}\right)\right)}{\sqrt{2-\left[\sin\left(\frac{\delta_x}{2}\right)\left(\cos(x')\cos\left(\frac{\delta_y}{2}\right)+\cos(x'-y')\sin\left(\frac{\delta_y}{2}\right)\right)+\cos\left(\frac{\delta_x}{2}\right)\left(-\cos\left(\frac{\delta_y}{2}\right)+\cos(y')\sin\left(\frac{\delta_y}{2}\right)\right)\right]^2}},
\end{aligned} \tag{S3}$$

where $x' = 2\pi x/\Lambda$ and $y' = 2\pi y/\Lambda$, with Λ the spatial period of the g-plates, corresponding to the first Brillouin zone of the simulated lattice model. For a given (δ_x, δ_y) setting, the Skyrme number is computed by evaluating the integral of Eq. (6) over a full spatial period.

* francesco.dicolandrea@unina.it

† filippo.cardano2@unina.it

S2. MACHINE-LEARNING ROUTINE

Following Ref. [1], our strategy is to train a feedforward neural network to predict the optimal $(E, \mathbf{n})$ parameters compatible with a set of experimental outcomes at each pixel. The training set consists of a suitable set of projective measurements, as prescribed by Eqs. (12)-(13), and the target outputs are directly related to the transformation parameters (E, n_x, n_y), while n_z is obtained from the normalization condition: $n_z = \pm\sqrt{1 - n_x^2 - n_y^2}$. The sign of the last component is determined by imposing continuity on the reconstructed map, after the network has output predictions over the entire pixel grid [1].

Specifically, we consider a fully-connected neural network, where each neuron in a given layer is connected to every neuron in the subsequent layer. We choose the Rectified Linear Unit ("ReLu") as the activation function for all the layers, except for the output layer, for which we choose the Sigmoid function. Remarkably, training data are generated at run-time. On the one hand, this reduces memory usage and computational requirements; on the other hand, it also helps to avoid *overfitting*, which might result from a too limited number of training samples. Several standard loss functions have been tested for supervised learning. Among these, the Mean Squared Error (MSE), evaluated directly on the process parameters, consistently showed the fastest convergence.

The learning process is divided into 50 epochs. During each epoch, the network explores a training set of 2^{20} randomly generated SU(2) processes, divided into 2^{12} *batches*. Each example in the training set thus corresponds to a single pixel in the optical experiment. At the end of each epoch, the performance of the network is evaluated on a different dataset, the *validation* set, consisting of 2^{18} processes, divided into 2^{10} batches. The validation phase works similarly to the training, but the weights are not adjusted after evaluating the cost function. To improve the learning performance, we also employ the callback "ReduceLROnPlateau". This function allows us to keep track of the loss during the various epochs and reduce the learning rate in case of stagnation, i.e., when the validation loss does not change appreciably over a given number of epochs, referred to as *patience*. To ensure good performance in the presence of noisy data, we employ Gaussian Dropout, in which noise is randomly applied to selected layers during training [2, 3]. Finally, the Adam optimizer [4] is used to adjust the network hyperparameters during supervised learning. The learning is realized by using the `TENSORFLOW` library [5].

For the sake of reproducibility, we report the complete setting of the neural-network hyperparameters in Table S1.

---

[1] F. Di Colandrea, L. Amato, R. Schiattarella, A. Dauphin, and F. Cardano, Retrieving space-dependent polarization transformations via near-optimal quantum process tomography, Opt. Express **31**, 31698 (2023).

[2] A. Labach, H. Salehinejad, and S. Valaee, Survey of dropout methods for deep neural networks, arXiv:1904.13310 .

[3] N. Srivastava, G. Hinton, A. Krizhevsky, I. Sutskever, and R. Salakhutdinov, Dropout: A simple way to prevent neural networks from overfitting, J. Mach. Learn. Res. **15**, 1929 (2014).

[4] D. P. Kingma and J. Ba, Adam: A method for stochastic optimization, arXiv:2204.00578 .

[5] M. Abadi, A. Agarwal, P. Barham, E. Brevdo, Z. Chen, C. Citro, G. S. Corrado, A. Davis, J. Dean, M. Devin, S. Ghemawat, I. Goodfellow, A. Harp, G. Irving, M. Isard, Y. Jia, R. Jozefowicz, L. Kaiser, M. Kudlur, J. Levenberg, D. Mané, R. Monga, S. Moore, D. Murray, C. Olah, M. Schuster, J. Shlens, B. Steiner, I. Sutskever, K. Talwar, P. Tucker, V. Vanhoucke, V. Vasudevan, F. Viégas, O. Vinyals, P. Warden, M. Wattenberg, M. Wicke, Y. Yu, and X. Zheng, TensorFlow: Large-scale machine learning on heterogeneous systems (2015), software available from tensorflow.org.

TABLE S1. Neural-network hyperparameters

Number of hidden layers	$N = 8$
Neurons in input layer	$N_{\text{in}} = 6$
Neurons in layer 1	$N_1 = 128$
Neurons in layer 2	$N_2 = 128$
Neurons in layer 3	$N_3 = 64$
Neurons in layer 4	$N_4 = 64$
Neurons in layer 5	$N_5 = 64$
Neurons in layer 6	$N_6 = 64$
Neurons in layer 7	$N_7 = 32$
Neurons in layer 8	$N_8 = 16$
Neurons in output layer	$N_{\text{out}} = 3$
Activations	
Layers 1, 2, 3, 4, 5, 6, 7, 8	ReLu
Output layer	Sigmoid
Data structure	
Batch size	$S_{\text{batch}} = 256$
Batches per epoch (training)	$N_T = 2^{12}$
Batches per epoch (validation)	$N_V = 2^{10}$
Number of epochs	$N_E = 50$
Optimization parameters	
Optimizer	Adam
Loss function	MSE
Learning rate (initial)	$\eta = 10^{-3}$
Gaussian Dropout rate	rate = 0.01
ReduceLROnPlateau:	
Patience	5 epochs
Reduction factor	$\mu = 0.1$
